# Optic nerve sheath diameter and spaceflight: defining shortcomings and future directions

**DOI:** 10.1038/s41526-022-00228-1

**Published:** 2022-10-06

**Authors:** Dylan A. Fall, Andrew G. Lee, Eric M. Bershad, Larry A. Kramer, Thomas H. Mader, Jonathan B. Clark, Mohammad I. Hirzallah

**Affiliations:** 1 Baylor College of Medicine and The Center for Space Medicine, Houston, TX USA; 2 Department of Ophthalmology, Blanton Eye Institute, Houston Methodist Hospital, Houston, TX USA; 3 Department of Ophthalmology, Cullen Eye Institute, Baylor College of Medicine, Houston, USA; 4 Department of Ophthalmology, Weill Cornell Medicine, New York, NY USA; 5 Department of Ophthalmology, University of Texas Medical Branch, Galveston, TX USA; 6 University of Texas MD Anderson Cancer Center, Houston, TX USA; 7 Texas A and M College of Medicine, Bryan, TX USA; 8 Department of Neurology, Baylor College of Medicine, Houston, TX USA; 9 Department of Diagnostic and Interventional Imaging, University of Texas Health Science Center, Houston, USA; 10 US Army, Moab, UT USA

**Keywords:** Research data, Eye diseases, Neurological disorders

## Abstract

Neuro-ocular changes during long-duration space flight are known as spaceflight-associated neuro-ocular syndrome (SANS). The ability to detect, monitor, and prevent SANS is a priority of current space medicine research efforts. Optic nerve sheath diameter (ONSD) measurement has been used both terrestrially and in microgravity as a proxy for measurements of elevated intracranial pressure. ONSD shows promise as a potential method of identifying and quantitating neuro-ocular changes during space flight. This review examines 13 studies measuring ONSD and its relationship to microgravity exposure or ground-based analogs, including head-down tilt, dry immersion, or animal models. The goal of this correspondence is to describe heterogeneity in the use of ONSD in the current SANS literature and make recommendations to reduce heterogeneity in future studies through standardization of imaging modalities, measurement techniques, and other aspects of study design.

## Introduction

Up to 60% of astronauts exposed to microgravity during long-duration space flight (LDSF) develop some degree of neuro-ocular changes^1^. These findings include optic disc edema, globe flattening, choroidal folds, hyperopic shifts in refraction, and optic nerve sheath distension. Together, these findings in astronauts have been termed “spaceflight associated neuro-ocular syndrome” (SANS). Although the specific etiology of SANS is unclear, possible etiologies include a cephalad fluid shift in microgravity that may cause chronically elevated intracranial pressure (ICP), cerebral venous congestion, lymphatic dysfunction, compartmentalization of cerebrospinal fluid (CSF) within the optic nerve sheath, disturbances in the one-carbon metabolic pathway, increased CO_2_ exposure, or a combination of these mechanisms^2^. Normal visual function is vital for mission performance and safety. Therefore, SANS is a crucial NASA research priority to further understand and prevent visual compromise during LDSF^3^. A portable, reliable, non-invasive measurement tool for evaluating the magnitude of optic nerve sheath expansion in SANS remains an area of need.

Optic nerve sheath diameter (ONSD) has been proven to reliably detect elevated ICP in terrestrial clinical studies^4–8^. In multiple meta-analyses and systemic reviews, increased ONSD has been found to be a highly sensitive (ranging from 90% to 93%) and moderately specific (ranging from 74% to 85%) metric for detecting elevated ICP^4,5^, with an overall mean ONSD difference of 1.3 mm (95% CI 0.6–1.9 mm) between elevated and normal ICP^7^. ONSD ultrasonography (US) is portable and non-invasive, making it ideal for use in evaluating unstable patients, in low resource settings^9^, and during space flight where a spectrum of ocular US measurements has been performed for years^10^. Magnetic resonance imaging (MRI) of the brain and orbits is also a part of the routine terrestrial pre- and post-space flight surveillance in astronauts, and while not portable, it remains a valuable tool to further our understanding of SANS^11^. Despite ONSD measurement’s potential benefits in detecting elevated ICP terrestrially and monitoring SANS manifestation during space flight, ONSD utilization is limited by variability in measurement technique and positive test cutoff values^6,12^. This variability highlights a need for standardization of ONSD measurement, technical specifications, and study design both for terrestrial and spaceflight use.

This article provides a focused qualitative review and discussion of ONSD use in current aerospace medicine literature from both ISS astronaut and ground-based analog studies, critically evaluates these studies, and attempts to uncover the current limitations of ONSD measurements in SANS-related research. Using this methodology, we intend to highlight shortcomings in current ONSD research and provide practical guidelines to standardize and improve the utility of ONSD measurement for the evaluation of SANS.

## Review

### Review methodology and overview

We assessed the current methods and measurement quality of ONSD in the literature by searching PubMed and Google Scholar for the following keywords: “optic nerve”, “optic nerve sheath”, “optic nerve sheath diameter”, “ocular”, “space flight associated neuro-ocular syndrome”, “visual impairment due to intracranial pressure”, “space flight”, and “microgravity”. Search terms were combined in a Boolean search format. Additional articles were identified by ancestry from relevant articles. Included manuscripts were limited to those that measured ONSD related to microgravity exposure or ground-based microgravity analogs; possessed qualitative or quantitative descriptions of the ONSD; were fully accessible peer-reviewed papers; and were published in English. The review was focused on experimental design, image acquisition, qualitative evaluation of published sample images, and summarizing the main ONSD-related results. Close attention was given to ONSD image acquisition, measurement, validation, and interpretation. These terms were defined as follows: image acquisition; obtaining ONSD images using US or MRI including the technical specifications and imaging conditions, measurement; accurately placing calipers on the image to obtain an ONSD measurement, validation; independent confirmation of the measurement by multiple experts, and interpretation; using ONSD measurements to interpret underlying pathology or physiology.

This review examined 13 studies that measured ONSD for the evaluation of the effects of microgravity or its analogs. Of those, 7 studies evaluated astronauts subjected to microgravity^1,11,13–17^, and 6 only evaluated subjects examined in various ground-based analogs designed to replicate certain aspects of microgravity^18–23^, with one study using both astronauts and ground-based analogs^17^. In the studies we examined, 6 measured ONSD using US^17–19,21–23^, 6 used MRI^1,11,13,14,16,20^, and one used both methods^15^.

### Qualitative evaluation of published sample images

**Fig. 1:**
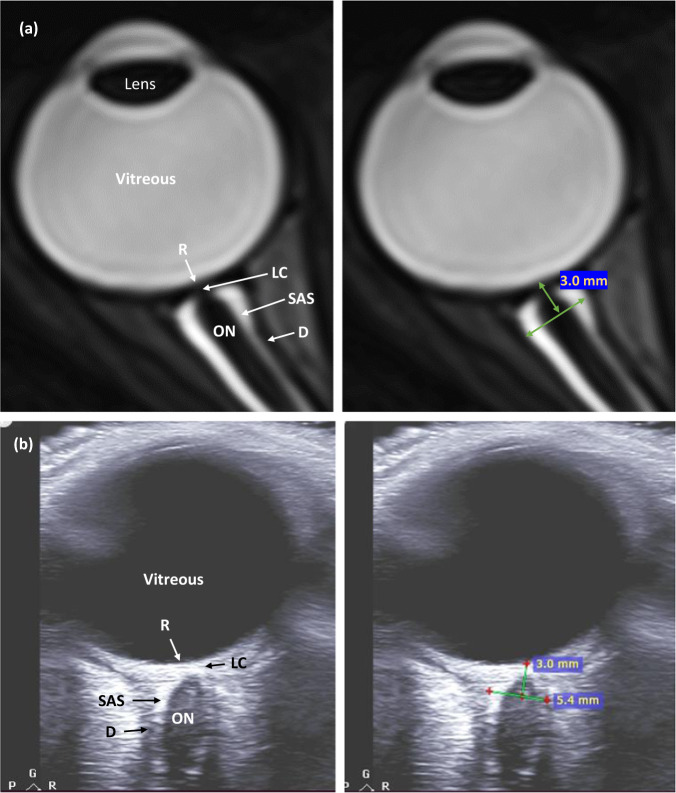
Example of anatomy and correct measurement of ONSD. Both MRI (**a**) and US (**b**) are represented in the figure. Note the measurement was conducted on a plane where the hypoechoic arachnoid and dural layer are clearly distinguished from the hyperechoic retroorbital face and subarachnoid space. R Retina, LC lamina cribrosa, ON optic nerve, SAS subarachnoid space, D dura.

There is no established consensus on ONSD image quality criteria. Suggested US ONSD quality criteria^24,25^ were modified to focus on the qualitative evaluation of published images as follows: (i) anatomic differentiation; good contrast between the posterior sclera, optic nerve, periorbital CSF, optic nerve sheath, and retrobulbar fat. (ii) Meningeal boundaries; clearly visible subarachnoid space, meningeal boundaries, and retrobulbar fat. (iii) Optic nerve head view; an ideal view represents the thinnest retinal–vitreous interface without the interposition of the thick sclera. (iv) Measurement depth; measurement should be performed at 3 mm posterior to the vitreous–retinal interface. (v) ONSD measurement; correct ONSD measurement is performed at the boundary of subarachnoid space and dura. These criteria were used for the evaluation of published sample US and MRI ONSD images, when available. Anatomic definitions and a measurement notation example are provided in Fig. 1.

Sample images were not published for 2 of the 13 studies included in this review^19,21^. The remaining 11 studies published samples including 5 of 7 studies using US^15,17,18,22,23^ and 7 of 7 studies using MRI^1,11,13–16,20^. Of the 11 studies with sample images, 3 had images that fulfilled all 5 ONSD quality criteria^11,15,22^, 10 demonstrated adequate anatomic differentiation with clear views of meningeal boundaries and optic nerve head^1,11,13–17,20,22,23^, 4 provided correct annotation of measurement depth on the sample image^11,15,18,22^, and 3 provided proper annotation of the ONSD measurement^11,15,22^. Table 1 is a summary of the qualitative image assessment and ONSD-related results of the included studies. The methods, objectives, and qualitative sample image evaluation of these studies are highlighted below.

**Table 1:** Characteristics, qualitative evaluation, and ONSD findings summary of included studies.

Study	Study type	Population (*N* and gender)	MRI or US	AD	MB	OV	MD	OM	Technical notes	Limitations	Main ONSD-related findings
Mader et al., 2013^15^	Case report	Astronaut (1M)	MRI	✔	✔	✔	✔	✔	Sagittal and axial T2-weighted sequence.		Case report of an astronaut with two LDSF (6 months each, 9 years apart). First flight measurements not performed. Second flight showed Increase of ONSD from ONSD range 6.2–7 mm on pre-flight US and MRI to 6.8–7.4 mm on inflight ultrasound. This persisted on post-flight US and MRI (0.73–0.75 mm).
Kramer et al., 2012^11^	Retrospective data analysis	Astronauts (27 NS), 8 underwent multiple missions	MRI	✔	✔	✔	✔	✔	3 T MRI Axial T2-weighted sequence with thin sections. 3D ONS sections are also used for measurement. right and left reported independently at 3, 4, and 5 mm depths.		Larger ONSD in astronauts with posterior globe flattening (mean ONSD of 7.2 ± 1.5 mm) compared to those without (mean ONSD of 5.8 ± 0.6 mm, *P* = 0.01). Similarly, astronauts with optic nerve sheath kinking had larger ONSD (7.5 ± 1.1 mm) compared to those without (5.9 ± 0.8 mm, *P* = 0.03). There were no significant differences in imaging for subjects with multiple spaceflights.
			US	✔	✔	✔		✔	13 MHz linear array probe, 1 axial view.		
Hamilton et al., 2011^22^	Experimental animal analog study	Adult pigs (5F)	US	✔	✔	✔	✔	✔	10 MHz linear array and 6–10 MHz curved array. 1 axial view.	Image quality assessment difficult in animal study, linear and curved transducers used interchangeably.	ONSD increased by 0.0034 mm ± 0.0003 per 1 mmHg increase in ICP, ranging from 0.0025 to 0.0046 for specific animals.
Mader et al., 2011^1^	Case series	Astronauts (7M)	MRI	✔	✔	✔			3 T MRI Axial T2-weighted sequence.	No ONSD measurements.	1 astronaut did not receive MRI, 3 had bilateral ONS distension, 2 had ONS distension right > left, 1 had isolated right ONS distension.
Mader et al., 2021^13^	Case series	Astronauts (3M)	MRI	✔	✔	✔			3 T Axial T2-weighted sequence.	No ONSD measurements in 2 astronauts.	Case 1: asymmetric ONS preflight (9.48 mm right and 6.00 mm left), increased ONSD following LDSF (9.93 mm right and 8.30 mm left) and, 7 years post-flight, ONSD returned to preflight values (9.47 mm right and 6.49 mm left) Case 2: “Mild ONS distension”, not quantified. Case 3: “Mildly dilated ONSs”, not quantified.
Mader et al., 2017^14^	Case report	Astronaut (1M)	MRI	✔	✔	✔			3 T MRI Axial T2-weighted sequence.	No ONSD measurements.	Bilateral ONS distension. “Distension” not quantified.
Rohr et al., 2020^16^	Longitudinal study	Astronauts (10 NS)	MRI	✔	✔	✔			3 T MRI, high-resolution, T1- weighted sagittal and T2-weighted coronal scans.	Automated method to measure ONS area not diameter. ONSD reported in one table, but measurement method not specified.	No significant changes in ONS area for most individuals after spaceflight and found that ONSD largely remained stable during the five post-flight recovery scans, from an average of 5.98 mm (5.40–6.51) preflight to 5.96 (5.36–6.49) R + 1, 6.04 (5.48–6.57) R + 30, 5.90 (5.31–6.44) R + 90, 6.01 (5.42–6.55) R + 180, 5.94 (5.34– 6.48) R + 360. Authors also noted no correlation between ONS kinking and ONS area (*R*^2^ = 0.22).
Marshall-Goebel et al., 2017^20^	Before and after HDT Study with countermeasures	Volunteers (9M)	MRI	✔	✔	✔			3 T Axial T2 MRI sequence. ONSD reported independently at 3, 4, and 5 mm depths.	ONSD averaged bilaterally.	Increased ONSD with steeper HDT angles most notable at the 3 mm depth compared to 4 and 5 mm depth. Average ONSD increased from 6 ± 0.6 mm (supine baseline) to 6.3 ± 0.5 mm (−6° HDT, *P* < 0.001), 6.3 ± 0.5 mm (−12° HDT, *P* < 0.001), and 6.5 ± 0.6 mm (−18° HDT, *P* < 0.001). Notably, the addition of −20 mmHg LBNP was able to reduce ONSD at all measurement points. The addition of 1% ambient CO_2_ during −12° HDT had no additional effect on ONSD.
Sirek et al., 2014^17^	Retrospective astronaut data + prospective HDT Study	Astronauts (17 NS) and Volunteers (6 NS)	US	✔	✔	✔			3–13 MHz and 8–12 MHz linear array probes, 1 axial view.	ONSD averaged bilaterally, providing mean differences rather than actual means, and 0.31 mm change is statistically significant	Retrospective astronaut data analysis: Combined right and left eye demonstrated an increase of 0.91 mm (*P* = 0.012) inflight compared to postflight, with resolution postflight. Prospective HDT study: Combined right and left eye ONSD increased an average of 0.31 mm from supine to 6-degree HDT (*P* < 0.0036), and 0.53 mm from supine to 30° HDT (*P* < 0.001).
Nusbaum et al., 2013^23^	Experimental Animal Analog Study	Juvenile pigs (30 NS)	US	✔	✔	✔			6–15 MHz linear array probe. Reported using coronal and sagittal views but sample image shows axial view.	Image quality assessment difficult in animal study. Reported only left eye findings. ONSD incorrectly included hypoechoic dura in venous congestion image but not normal image; possibly falsely increased ONSD.	The group with restricted cerebral venous flow had a significant increase in CVP, ICP, and ONSD compared to placebo (ONSD change from 4.4 ± 0.4 mm at baseline to 6.5 ± 0.6 mm at 3 h, *P* = 0.0001).
Kondrashova et al., 2019^18^	Before and after HDT Study	Volunteers (21M, 11F)	US				✔		5–12 MHz linear array probe, 1 axial view.	Inaccurate measurement, HDT angle NS, and ONSD averaged bilaterally.	Increase in ONSD from pre-inversion (5.02 ± 0.05 mm) to inversion (6.04 ± 0.08 mm, *P* < 0.001) and to post-inversion (6.36 ± 0.08 mm, *P* < 0.001).
Laurie et al., 2017^19^	Before and after HDT Study	Volunteers (8M)	US	NA	NA	NA	NA	NA	12 MHz linear array probe	No published US image. Did not specify measurement depth or structures measured.	Increase in ONSD between supine (6.23 ± 0.52 mm) and both the 6° HDT and 6° HDT + CO_2_ conditions (6.58 ± 0.52 mm and 6.66 ± 0.52 mm, respectively, *P* < 0.05 for both) but did not find any significant changes between 6° HDT and 6° HDT + CO_2_ (*P* > 0.05).
Kermorgant et al., 2017^21^	Before and after Dry Immersion Study	Volunteers (12M)	US	NA	NA	NA	NA	NA	ONSD was average of the four measures: horizontal and vertical bilaterally. Expert performing measurement blinded to experimental condition.	No published US image. Did not specify measurement depth or structures measured. ONSD averaged bilaterally.	Increased ONSD compared to baseline during the dry immersion, with an average baseline of 4.64 ± 0.4 mm, then 5.94 ± 0.67 mm on Day 1, and 6.01 ± 0.49 mm on Day 3. Subjects stratified into “poor recovery” if ONSD increases >20% from baseline to post-test (average of 6.16 ± 0.62 mm post-test) or “good recovery” group (average ONSD 5.17 ± 0.47 mm post-test). The “good recovery” group had better autoregulation after dry immersion than the “bad recovery group” as measured by a transcranial doppler autoregulation index.

*CVP* central venous pressure, *F* Female, *HDT* head down tilt, *ICP* intracranial pressure, *LBNP* lower body negative pressure, *MRI* magnetic resonance imaging, *M* Male, *NS* not specified, *ONH* optic nerve head, *ONS* optic nerve sheath, *ONSD* optic nerve sheath diameter, *R*+ *return day*, *T* Tesla, *US* ultrasonography. Qualitative image analysis: *AD* anatomic differentiation, *MB* meningeal boundaries, *OV* Optic nerve head view, *MD* measurement depth, *OM* ONSD measurement, ✔: meets qualitative image evaluation criteria, *NA* not available.

### Astronauts exposed to microgravity

Mader et al. in 2011 reported optic nerve sheath (ONS) distention in 6 of 7 astronauts that underwent MRI imaging following LDSF^1^. Notably, ONS distension was mostly asymmetric in 3 of the 6 astronauts. In 2021, Mader et al. followed 3 of the astronauts from the original 2011 case series who had persistent globe flattening for 7 years or more following LDSF^13^. In 2017, Mader et al. described another astronaut with asymmetric optic disk swelling and increased ONSD in both eyes after LDSF^14^. Strengths: all three studies had images with good anatomic differentiation, meningeal boundaries, and optic nerve head views. Weaknesses: not reporting ONSD measurement or measurement depth. Quantitative ONSD measurements were not reported in the 2011 and 2017 publications, and in only one subject in the 2021 publication, limiting the ability to compare these findings to more recent literature.

The Mader et al. 2013 case study of an astronaut with two LDSFs (6 months each, 9 years apart) provided measurements of the ONSD taken by both MRI and US^15^. All recorded measurements were for the second flight, and MRI measurements were taken 9 months pre-flight as well as 6 days post-flight, while the US was done 11 and 6 months pre-flight; 1, 3, and 5 months inflight; and 3 days post-flight. The first flight occurred prior to the implementation of in-flight ocular ultrasound monitoring and images are not available for comparison^15^. Strengths: the study provided quantitative measurement of the ONSD that demonstrated an increase in US ONSD from baseline MRI and US ONSD which persisted 3–6 days post-flight on both US and MRI; the MRI images from this study met all quality criteria with good anatomic differentiation, meningeal boundaries, optic nerve head views, measurement depth and accurate placement of ONSD measurement markings; the US images demonstrated good anatomic differentiation, meningeal boundaries, optic nerve head views, and accurate placement of ONSD measurement markings. Weaknesses: this study did not specify the measurement depth on ultrasound images.

Kramer et al. in 2012 retrospectively reviewed MRI studies of 27 astronauts after LDSF. Eight of these astronauts had MRI studies done before and after an additional spaceflight^11^. Strengths: the MRI images from this study met all quality criteria with good anatomic differentiation, meningeal boundaries, optic nerve head views, measurement depth, and accurate placement of ONSD measurement markings; ONSD of the right and left eyes were measured independently at three depths (3, 4, and 5 mm from the vitreoretinal interface); the measurements were done by two experienced radiologists and repeated by one reader, demonstrating high inter- and intra- observer reliability. No weaknesses were identified. This study showed that astronauts with ONS kinking or globe flattening had larger ONSD measurements, but no significant association was found between the number of spaceflights and ONSD.

Rohr et al. used MRI combined with semi-automated image analysis to assess ocular changes in 10 astronauts with prior microgravity exposure who underwent additional missions^16^. MRI studies performed preflight and at 5 timepoints post-flight, extending as far as 1 year, were reported. The authors manually marked the optic nerve head, then used automated image analysis to analyze the boundary contours of a coronal ONS slice 3 mm posterior to the optic nerve head to assess the optic nerve sheath cross-sectional area. Strengths of this study included MRI images with good anatomic differentiation, clear meningeal boundaries, and optimal ONH view; measurement depth of 3 mm from the ONH; and accurately marking of the meningeal boundaries for the ONS area. Weaknesses: lack of manual measurements to validate the automated technique; ONSD was reported in one table and measurement methods were not specified; and the measurement depth and ONSD were not marked on an axial image to allow for qualitative evaluation.

Sirek et al. used US to study ONSD and central retinal artery blood flow before, during, and after microgravity exposure in astronauts and compared these changes with those found in microgravity-naïve healthy volunteers subjected to 20 min of head-down tilt (HDT) at 6° and 30°^17^. Strengths: US images demonstrated good anatomic differentiation, meningeal boundaries, and ONH view; ONSD measurement depth of 3 mm from the vitreoretinal interface on the inner dural margin. Weaknesses: lack of markers on the sample US image to allow qualitative evaluation; ONSD was averaged bilaterally, which may mask ONSD changes given ONSD asymmetry in SANS^1,13^; and using mean differences rather than actual means which limits the interpretation of findings in the context of other literature.

### Ground-based analogs—human studies

Kondrashova et al. measured changes in ONSD using the US before, during, and after a 3-min inversion in the Trendelenburg position^18^. These measurements were performed by medical students under the supervision of a physician experienced in ultrasound. Strength: accurate ONSD depth markers placement at the vitreoretinal interface. Weaknesses included lack of clear anatomic differentiation, clear meningeal boundaries, or a consistently clear view of the ONH, as one of the published images, showed a thick scleral band obstructing the ONH; the ONSD markers appear inaccurate given the poor anatomic differentiation; the study reported combined eye ONSD findings rather than report each eye independently; the authors did not specify the HDT angle, which is important to report as steeper angles cause a higher ONSD^17,20^; the unblinded nature of the study combined with unclear meningeal boundaries may have contributed to errors through expectancy bias and measurement errors favoring artificially larger measurements after HDT.

Laurie et al. in a 6° HDT for 1 h study had a similar design as Kondrashova et al. with the additional variable of 1% inhaled CO_2_^19^. Strength: analysis of ultrasound images by at least two sonographers. Weaknesses: lack of details on ultrasound technique and no sample images were published to verify the quality of measurements.

Marshall-Goebel et al. performed an HDT + CO_2_ study that evaluated a lower body negative pressure (LBNP) device as a potential SANS countermeasure^20^. Orbital MRI was used to evaluate ONSD changes after 5 h of different levels of HDT (−6°, −12°, and −18°), −12° HDT with 1% inhaled CO_2_, and −12° HDT while wearing a −20 mmHg LBNP device as a potential countermeasure to cephalic fluid shift. Strengths: MRI images demonstrated good anatomic differentiation, clear meningeal boundaries, and optimal ONH view; measurements were performed at the correct depth and checked for accuracy by an additional expert observer; and ONSD measurements were performed bilaterally. Weaknesses: right and left eye ONSD measurements were averaged together; vague depth reference structure “behind the eye globe”; and no depth or ONSD markings were annotated on the sample image, preventing qualitative evaluation of measurement technique.

Kermorgant et al. used dry immersion to simulate weightlessness in healthy volunteers for 3 days^21^. Subjects were placed supine in a thermoneutral water bath, with a layer of fabric separating the subject from the fluid to simulate the weightlessness and cephalad fluid shift that occurs in microgravity. ONSD was measured at baseline before the study, on days 1 and 3 of dry immersion, and 1 day after dry immersion. Strengths: ONSD depth measurement at 3 mm from the vitreoretinal interface; two views (transverse and sagittal plane) were taken for each eye; each measurement was validated by an expert observer, and the expert performing the measurements was blinded to the experimental conditions, which increases the internal validity of the study findings. Weaknesses: lack of published sample images for qualitative evaluation and the vague depth reference structure “behind the eye globe”; and averaging ONSD measurements bilaterally.

### Ground-based analogs—animal studies

Hamilton et al. investigated the relationship between ICP and ONSD in a porcine animal study^22^. While measuring ONSD at the 3–5 mm depth via ultrasound, saline was infused into the brain parenchyma to artificially increase ICP and identified a positive and direct relationship between ICP and ONSD. IV saline bolus was used to increase central venous pressure (CVP) in one animal. There was a clear relationship between ICP and CVP, but there was a lack of a relationship between ONSD and CVP. Strengths: ONSD images demonstrated good anatomic differentiation, clear meningeal boundaries, and optimal ONH view; appropriate depth and ONSD marker placement; and eye measurements were reported independently. The weakness of this paper was the use of a curvilinear US probe on some of the images used, rather than the linear probe typically used for ONSD measurements.

Nusbaum et al. further studied the effect of cerebral venous congestion on ICP and ONSD in an animal model^23^. Using juvenile porcine subjects, superior vena cava balloon catheters were inflated to induce restricted cerebral venous outflow. Changes in internal vena cava pressure, external vena cava pressure, ICP, and ONSD were monitored. The group with restricted cerebral venous flow had a significant increase in CVP, ICP, and ONSD compared to the placebo. Strengths: images demonstrated good anatomic differentiation, meningeal boundaries, and ONH views. Weaknesses: lack of depth measurement markers, and no clear description of the depth reference structure; the ONSD measurements incorrectly included the hypoechoic dura in the measurement for the venous congestion image but not the normal image, raising concerns that the experimental group had artificially higher ONSD values; the study reported that the measurements were performed in the coronal and sagittal views while the sample image shows an axial view; and ONSD measurements were performed at the 2 mm depth, which is different from the standard 3 mm depth used in humans.

### Summary of current literature results

ONSD was used for the evaluation of SANS and its analogs in astronauts, healthy volunteers, and animal models. Of the 7 astronaut studies, 2 demonstrated significant US ONSD increase during spaceflight^15,17^, 3 showed a persistent and significant change in MRI ONSD after spaceflight^11,13,15^, 3 showed a qualitative increase in MRI ONSD without providing quantification^1,13,14^, and one study showed no change in ONSD on MRI after spaceflight^16^. Of the four studies using ground-based analogs, 3 measured the ONSD with US^17–19,21^, and one study used MRI^20^. All ground-analog studies found significant increases associated with the cephalad fluid shift induced by HDT^17–20^ or dry immersion^21^. Other interesting observations include a significant decrease in ONSD associated with lower body negative pressure (LBNP)^20^, no significant ONSD change with elevated CO_2_ levels and HDT^19,20^, and the association between <20% increase in ONSD with better cerebral autoregulation during dry immersion when compared to subjects with >20% ONSD change^21^. Two animal studies used the US to illustrate a direct, positive relationship between ICP, CVP, and ONSD to various degrees^22,23^.

### Defining shortcomings of current literature

A review of these studies demonstrated variations in metrics used for ONS evaluation including qualitative ONS distension, ONS area, and ONSD, variations in measurement depth and structures included, and variations in MRI sequence or US probe transducers used. We summarize these findings, limitations, and proposed solutions in Table 2.

**Table 2:** Findings and limitations of current literature and proposed solutions.

Findings	Limitations	Recommendations
MRI images were obtained before and after flight^1,13–16^ with US images mostly obtained inflight^15,17^	It may be difficult to compare US and MRI ONSD measurements that are not obtained at the same time.	Consider obtaining images with both MRI and US at baseline to verify consistency across modalities.
Using a curvilinear ultrasound probe^22^	It is difficult to compare results between studies if researchers are using different probe types and settings. Linear probes are preferred for ONSD measurements^24,25^.	Linear ultrasound probe with appropriate ocular settings should be used for ONSD measurement.
No sample image of measurement method provided^19,21^	Difficult to evaluate ONSD measurement quality without a sample image.	Given the large heterogeneity in ONSD measurements^5,8,40^, it is important to provide a sample image for qualitative assessment.
No annotation of measurements on sample images^1,13,14,16–18,20,23^	Difficult to evaluate ONSD measurement depth and structures included without annotation on a sample image.	Sample images should include annotation of depth used and structures included in the measurement.
Unclear measurement depth^1,13,14,19,21^ or non-standard depth^23^ recorded	ONSD is measurement can change at different depths.	The measurement should be performed 3 mm from the ONH at the vitreoretinal interface. This should be clearly documented and annotated on the sample published image.
Sample image lacks anatomic differentiation (contrast) needed to accurately measure ONSD^18^	Risk for under or overestimation of ONSD.	Image acquisition should provide clear anatomic differentiation of the ONS.
Inclusion of the dura in ONSD measurement^23^	Overestimation of ONSD.	ONSD measurement should start at the interface between the Subarachnoid space (hyperechoic on US and hyperintense on T2 MRI) and the dura (Hypoechoic on US and Hypointense on T2 MRI).
Studies combined left and right eye measurements^17,18,20,21^ or did not specify right vs. left or combined^16,19^	ONSD asymmetry has been documented in astronauts with SANS^14^. Since the presence of findings unilaterally is consistent with SANS under the current working definition^41^, averaging asymmetric measurements may lead to underestimating ONSD and missing SANS manifestation.	Eye findings should be reported independently for each eye particularly when diagnostic thresholds are being evaluated or considered.
Reporting means differences in ONSD measurements without reporting actual pre-, in-, and post-flight ONSD means^17^	Unable to compare ONSD values to other published values in the literature.	Values for each experimental position or mission profile should be reported and not only mean differences.
Researchers performing measurements were not expertly trained^18^ or blinded to the subject’s condition^18,23^ leading to overestimation of ONSD measurement in the experimental group	Measurement bias combined with poor anatomic differentiation of acquired image or limited training may lead to falsely elevated ONSD findings in the experimental group.	When possible, measurement for research purposes should be performed by an expert with adequate training that is blinded to the condition under which the image was obtained^21^.
Inflight US ONSD measurements are performed routinely in many astronauts for SANS surveillance^42^. However, findings are published on only 18 astronauts^15,17^	Publication gap limits investigators understanding of the effects of spaceflight on ONSD.	Perform a retrospective analysis of available astronaut inflight US ONSD data.

ONSD can be evaluated with MRI or US, and each modality has advantages and disadvantages. The main advantages of MRI are lower vulnerability to user error for image acquisition, excellent soft tissue contrast, visualization of the entire optic nerve, and the ability to utilize automated quantification and three-dimensional reconstructions. MRI use is limited by high cost, lack of portability, and current inability to perform during spaceflight. In contrast, US ONSD measurement is a highly portable, inexpensive, and dynamic methodology that can be conducted quickly at virtually any location. This versatility comes with the limitation of needing an expert operator to obtain high-quality images and perform the measurements. Astronauts on the ISS have successfully performed the ocular US with remote guidance and have obtained good quality US images^26^. While MRI and US measurements of the same subject at the same time point showed excellent consistency^27^, it is not possible to use this data from different SANS ONSD studies interchangeably due to the variability in timing and measurement methods. MRI and ultrasound are used at different time points to measure ONSD primarily due to logistical considerations. The inability to use MRI to measure ONSD during spaceflight limits the utility of MRI studies since ONSD changes rapidly as demonstrated by head-down tilt studies, which measured significant changes after 3 min–5 h of HDT^18–20^ and while undergoing lumbar puncture in terrestrial settings^28^. This rapid change in ONSD diameter may lead to underestimation of ONSD changes as it may have returned to its preflight value before the opportunity for postflight measurement. Therefore, the only changes that would be detected by MRI postflight would be those where the extent of distension caused long-term structural changes in the ONS^29^ which may persist for years after LDSF^13–15^. This behavior, known as hysteresis, occurs when ONSD reversibility may be impaired after episodes of prolonged high pressures within the ONS^30^. Therefore, it may be useful to use both MRI and US at baseline to verify consistency between modalities.

Issues that may limit ONSD image acquisition and measurement in SANS studies included lack of sample images in publications^19,21^ to confirm image quality, lack of annotation of measurements on sample images^1,13,14,16–18,20,23^ to verify measurement of correct structures, unclear measurement depth^1,13,14,19,21^ or using non-standard depth^23^, sample images lacking anatomic differentiation (contrast) needed to accurately measure ONSD^18^, using a curvilinear ultrasound probe^22^ as opposed to the standard of using a linear probe, and inclusion of the dura in ONSD measurement^23^ as opposed to the measurement at the interface between the SAS and dura. These findings, limitations, and proposed solutions are discussed in Table 2.

Challenges that limited ONSD reporting and interpretation in SANS studies included combining left and right eye measurements^17,18,20,21^ or not specifying whether the measurements were combined^16,19^, reporting differences in mean in ONSD measurements without reporting actual pre-, in-, and post-flight means^17^, limited experience in ONSD measurement^18^, and lack of blinding potentially leading to overestimation of ONSD measurement in the experimental group^18,23^. These findings, limitations, and proposed solutions are discussed in Table 2.

Inflight US ONSD measurements are performed routinely in many astronauts for SANS surveillance^31^. However, inflight ONSD findings are published in only 18 astronauts^15,17^. This publication gap limits the development of future research projects that better utilize US ONSD for the inflight evaluation of SANS, and publishing this data should be a research priority.

An additional gap in knowledge that limits the interpretation of ONSD in the evaluation of SANS is the lack of a clear cutoff to distinguish “normal” from “pathologic”. In the terrestrial setting, the cutoff used to detect elevated ICP has also varied between trials in the range of 5.0–6.4 mm^25,32^. These studies are not only limited by the large range but also cannot directly carry over to SANS, which is a different pathologic process with no direct evidence of ICP elevation in astronauts. While the currently published data on astronauts and ONSD is limited in quality and quantity, available spaceflight data suggests that ONSD increases with spaceflight, which warrants further research. Further study is also needed to establish the ONSD change that is appropriate for the detection of SANS. This gap in knowledge can be partially explained by the small sample size of astronauts with ONSD measurements available for study^2^. It should be noted that while the clinical and imaging characteristics of SANS have been empirically described and revised periodically, there is no gold standard test for assessment of increased ONSD in SANS. Also, a certain degree of ONS distension could be protective for astronauts during spaceflight, allowing for greater levels of elasticity during cephalic fluid shifts^31^. Thus, questions remain unanswered regarding how much ONSD change one should expect during spaceflight, and what amount of ONSD increase can be considered pathological. Concurrent examination of optic disc edema in individual astronauts may help determine pathological thresholds of ONS expansion. Optic disc edema can be evaluated using techniques such as Optical Coherence Tomography and fundus photography, both of which are done on ISS astronauts in-flight. The correlation of venous congestion and US internal jugular vein cross-sectional area might also be useful, as this is also an ISS astronaut's in-flight capability.

### Future directions and proposed ONSD evaluation checklist

Addressing the publication gap by publishing inflight US ONSD data and correlating it with fundoscopy and venous distension, in addition to standardization of research methods and following clear quality criteria for ONSD image acquisition and measurement are essential for the continued utilization of ONSD in the evaluation of SANS.

There are no published consensus ONSD measurement criteria. The standardized checklist recommended in Table 3 is informed by suggested criteria^24,25,33,34^, the authors’ expert opinion, and concurrent work that is aiming to standardize ONSD measurement^12^.

**Table 3:** Recommended standardized checklist.

Imaging modality	Ultrasound	MRI
Probe selection (ultrasound) Coil selection and field strength (MRI)	• Linear array probe • Ocular setting • ≥7.5 MHz effective imaging frequency	• ≥3 T field strength • ≥32 channel head coil
Safety	• Avoid excessive globe pressure • Thermal index (TI) ≤ 1.0 • Mechanical index (MI) ≤ 0.23	• Standard MRI safety precautions
Positioning	• Supine position with no head of bed elevation • Neutral gaze to avoid ONS deformation with lateral gaze. • Probe: Lateral axial view recommended as a minimum. If other views obtained, they should be obtained in addition to this standard view.	• Supine position with neutral gaze
Image acquisition	• Avoid lens, if possible. • Adjust depth, focus, and gain to obtain good differentiation between ON (hypoechoic), ONS (hyperechoic), Dura (hypoechoic), and retro-orbital fat (hyperechoic) (see Fig. 1). • Image should be orthogonal to the optic nerve sheath, i.e., acquisition plane should be parallel to the nerve axis. • The image with the thinnest ONH where the hypoechoic ON meets the hypoechoic vitreous is the optimal view for measurement.	• Fat suppressed 3D T2-weighted sequence with ≤0.8 mm isotropic voxels acquired orthogonal to the optic nerve sheaths.
Measurement	• Depth: depth should be measured parallel to the ON axis starting at the vitreoretinal interface of the papilla. • ONSD: ONSD should be measured at the 3 mm depth perpendicular to the depth line starting at the interface between the hyperechoic ONS and hypoechoic dura (inside the dura). • When ONSD is used in research, the expert performing the measurement should be blinded to the conditions under which the image was acquired to prevent bias in measurement.	• Same as ultrasound for blinding, depth, and measurement axis. • ONSD should be measured at the interface between the hyperintense ONS and hypointense dura (inside the dura). • Same as ultrasound
Reporting findings	• Findings should not be averaged across right and left eyes given the asymmetry of SANS. • If ONSD is used for the diagnosis of SANS, the side with the larger ONSD should be used for analysis and the same side should be tracked over time to monitor progression. This recommendation may or may not apply to terrestrial medicine depending on the symmetry of the underlying disease process.	• Same as ultrasound
Interpretation of ONSD measurement	• Cut-offs for diagnosing elevated ICP or SANS are not currently well-established and further research is needed.	• Same as ultrasound

US ONSD image acquisition should utilize a high-frequency (commonly 12–14 MHz) linear array probe with ocular settings if available. For safety, excessive globe pressure should be avoided and a thermal index (TI) ≤ 1.0 and mechanical index (MI) ≤ 0.23 should be utilized. The subject should maintain a neutral gaze to avoid ONS deformation with lateral gaze and stay in a supine position unless the study specifically aims to study the effects of different body positions on ONSD. Lateral axial view is recommended as a minimum. If other views are obtained, they should be obtained in addition to this standard view.

US images should demonstrate good sonographic differentiation where the subarachnoid space can be clearly differentiated from surrounding structures and include a view of the ONH without a thick scleral band interposing between the retina and the optic nerve. Measurement should be performed at the 3 mm depth from the vitreoretinal interface, perpendicular to the nerve axis, and at the inner diameter of the dural sheath, the interface between the hypoechoic dural sheath and hyperechoic CSF in the SAS (Fig. 1, Table 3)^24,25^.

MRI measurement of ONSD can provide clearly defined margins between the high signal intensity vitreous fluid and CSF in the SAS, and the much lower signal intensity sclera and optic nerve sheath on fluid-sensitive T2-weighted sequences. MRI ONSD image acquisition parameters that could improve the accuracy of OSND measurements using this property of MRI are as follows: (i) maximize T2-weighted contrast between fluid and soft tissue structures using echo-times at 100 ms or greater. (ii) Acquisition of isotropic voxel size of 0.8 mm or less using a 3D T2-weighted sequence to minimize partial volume artifacts and increase edge detection of structures. Isotropic voxels also allow optimal orthogonal reconstruction of the optic nerve sheath relative to the optic disc improving cross-sectional measurement of the OSND. (iii) Image acquisition at 3 T or greater field strength with 32-channel or greater head coil to improve signal-to-noise of the small voxel size acquisition. (iv) Minimize chemical shift artifact which can blur margins of the optic nerve sheath from adjacent orbital fatty tissue using a fat suppression technique^33^. Similar to ultrasound, measurement should be performed at the 3 mm depth from the vitreoretinal interface, perpendicular to the nerve axis, and at the inner diameter of the dural sheath, the interface between the hypointense dural sheath and hyperintense CSF in the SAS (Fig. 1, Table 3)^16,35–39^.

## Outlook and summary

Addressing the inflight ONSD publication gap and standardization of ONSD image acquisition and measurement are essential to improve the utility of ONSD in SANS evaluation. This and future work on ONSD in SANS will be important in anticipation of future manned exploration of space.

### Reporting summary

Further information on research design is available in the Nature Research Reporting Summary linked to this article.

## Supplementary information

Reporting Summary

## Data Availability

All data generated or analyzed during this study are included in this published article.
